# Virtual reality improves the accuracy of simulated preoperative planning in temporal bones: a feasibility and validation study

**DOI:** 10.1007/s00405-020-06360-6

**Published:** 2020-09-22

**Authors:** Tomi Timonen, Matti Iso-Mustajärvi, Pia Linder, Antti Lehtimäki, Heikki Löppönen, Antti-Pekka Elomaa, Aarno Dietz

**Affiliations:** 1 Department of Otorhinolaryngology, Kuopio University Hospital, Puijonlaaksontie 2, PL 100, 70210 Kuopio, Finland; 2 Microsurgery Centre of Eastern Finland, Kuopio, Finland; 3 Department of Radiology, Kuopio University Hospital, Kuopio, Finland; 4 School of Medicine, Institute of Clinical Medicine, University of Eastern Finland, Kuopio, Finland

**Keywords:** Virtual reality, Two-dimensional, Three-dimensional, Surgical planning, Temporal bone

## Abstract

**Purpose:**

Consumer-grade virtual reality (VR) has recently enabled various medical applications, but more evidence supporting their validity is needed. We investigated the accuracy of simulated surgical planning in a VR environment (VR) with temporal bones and compared it to conventional cross-sectional image viewing in picture archiving and communication system (PACS) interface.

**Methods:**

Five experienced otologic surgeons measured significant anatomic structures and fiducials on five fresh-frozen cadaveric temporal bones in VR and cross-sectional viewing. Primary image data were acquired by computed tomography. In total, 275 anatomical landmark measurements and 250 measurements of the distance between fiducials were obtained with both methods. Distance measurements between the fiducials were confirmed by physical measurement obtained by Vernier caliper. The experts evaluated the subjective validity of both methods on a 5-point Likert scale qualitative survey.

**Results:**

A strong correlation based on intraclass coefficient was found between the methods on both the anatomical (*r* > 0.900) and fiducial measurements (*r* > 0.916). Two-tailed paired t-test and Bland–Altman plots demonstrated high equivalences between the VR and cross-sectional viewing with mean differences of 1.9% (*p* = 0.396) and 0.472 mm (*p* = 0.065) for anatomical and fiducial measurements, respectively. Gross measurement errors due to the misidentification of fiducials occurred more frequently in the cross-sectional viewing. The mean face and content validity rating for VR were significantly better compared to cross-sectional viewing (total mean score 4.11 vs 3.39, *p* < 0.001).

**Conclusion:**

Our study supports good accuracy and reliability of VR environment for simulated surgical planning in temporal bones compared to conventional cross-sectional visualization.

**Electronic supplementary material:**

The online version of this article (10.1007/s00405-020-06360-6) contains supplementary material, which is available to authorized users.

## Introduction

One key issue for successful and safe surgery is thorough preoperative planning. The foundation for detailed preoperative planning of surgical procedures is based on individual image data and should be considered mandatory for modern operative care. In otolaryngology, preoperative imaging is mostly performed with computed tomography (CT) and magnetic resonance imaging (MRI). The image-stack manipulation and measurements are usually based on picture archiving and communication system (PACS) tools and volume renderings. These limit the image view to two-dimensional (2D) cross-sections and screens, missing stereoscopy and freedom of dimensional control. Despite the advances in preoperative imaging, images are still examined in 2D cross sections thus requiring the surgeon to construct and formulate an understanding of complex three-dimensional relationships. This is challenging not only for novice surgeons but also for experienced surgeons in complex cases. Recent advances in virtual reality (VR) technologies have yielded numerous VR applications for surgical planning, which may overcome these restrictions [1]. These new VR applications offer promising tools for surgical training and preoperative planning [2–4].

The first medical VR applications were introduced in the 1990s. They mainly focused on visualization of complex anatomy, preoperative planning, surgery training and telemedicine [5]. In recent years, the availability of consumer-grade VR technology has re-emerged the interest for medical use. The VR is a stereoscopic three-dimensional (3D) computer-generated environment, which provides an interactive stereoscopic 3D view of objects. The development of head-mounted displays and hand-held controllers with motion tracking sensors provide users with a versatility and a possibility to approach the multidimensional anatomy of the patient at any possible angle. Users can freely control magnification, windowing of image parameters and mark or paint objects in the image view. State-of-the-art VR systems already reproduce 3D anatomy to a high level of immersion and authenticity not achievable with conventional cross-sectional 2D images and thus may contribute to a better understanding of the anatomy in question [6]. The VR environment allows users to perceive critical anatomical landmarks and their relationship in the same virtual space, which adds to better memory recall compared to traditional 2D screen interface [7]. However, nausea, vertigo and headache have been reported with VR in 30–80% of the users, depending on the software [8, 9].

VR surgical planning is gaining increasing attention since it has been shown to augment operative accuracy, efficiency and outcomes [10–12]. However, there are only very few independent studies, which investigate the validity and accuracy of VR in authentic settings [13]. Best practice requires that new medical applications such as the VR surgical planning software are tested for subjective and objective validity [14]. Subjective validity is commonly evaluated with the face and content validity via different acceptance surveys by experts in the field. The face validation demonstrates the degree of resemblance between a method under investigation and real activity. The content validity is established by demonstrating that the system or method measures what it is intended to measure in terms of e.g. surgery or planning [15–17].

Temporal bone (TB) and skull base anatomy are considered among the most complex anatomical regions in humans and their accurate evaluation and understanding is challenging even for experienced otologic and skull base surgeons. The aim of this study was to examine the accuracy of VR compared to cross-sectional viewing and to establish its feasibility in a simulated preoperative planning setting. Our hypothesis is that the accuracy of the VR environment is comparable to cross-sectional PACS viewing: however, it can help providing more accessible information on the topographical anatomy in TBs with better subjective validity compared to cross-sectional viewing.

## Materials and methods

The study had an institutional approval (No. 125/2019) and the National Supervisory Authority for Welfare and Health authorized the use of cadaveric TBs (No. 9202/06.01.03.01/2013). The study fulfilled the Helsinki Declaration for Ethical use of human material.

**Fig. 1:**
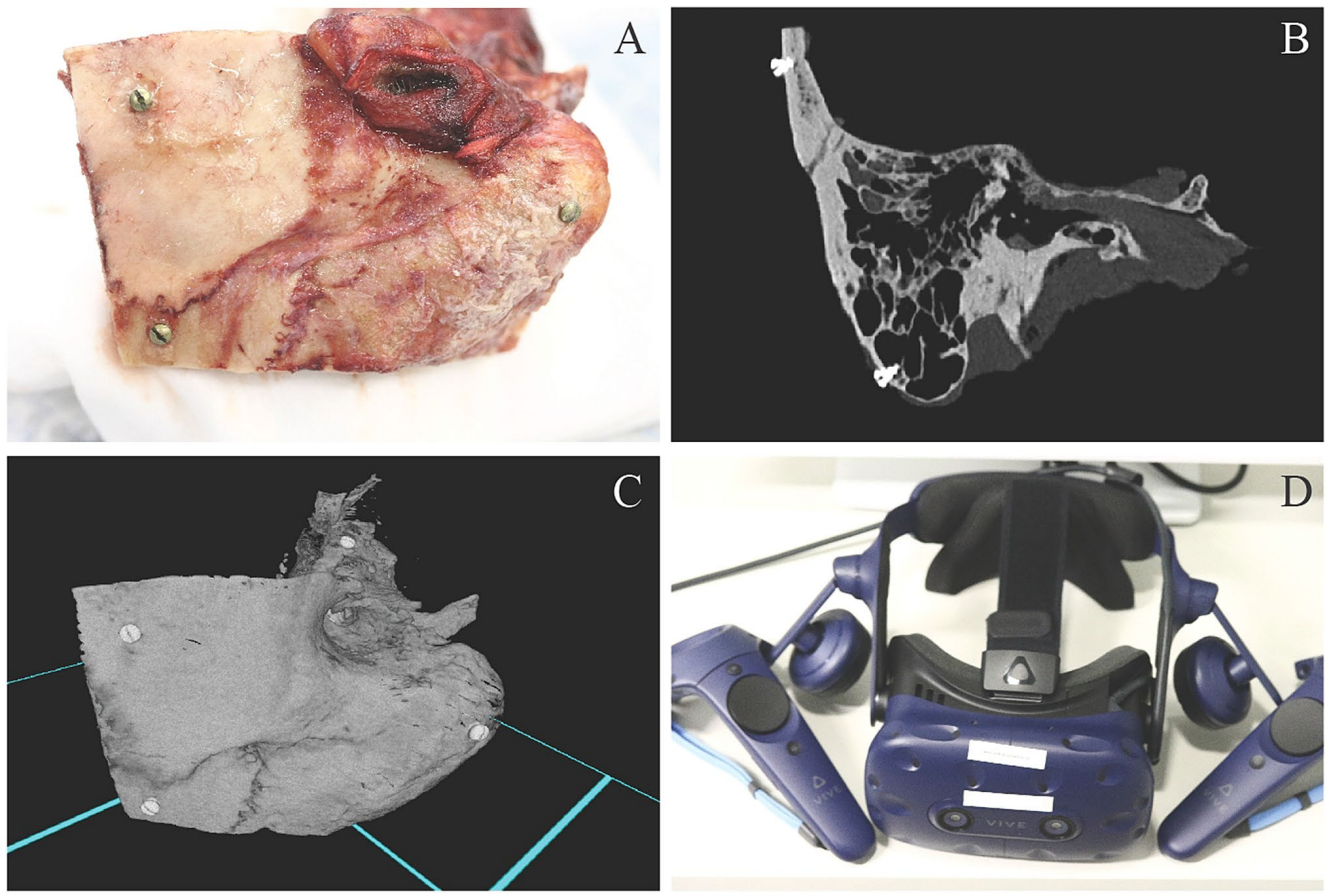
**a** Fresh frozen temporal bone, **b** Conventional 2D HRCT image of temporal bone, **c** Temporal bone in VR environment, **d** HTC Vive Pro head-mounted display and a pair of controllers

**Fig. 2:**
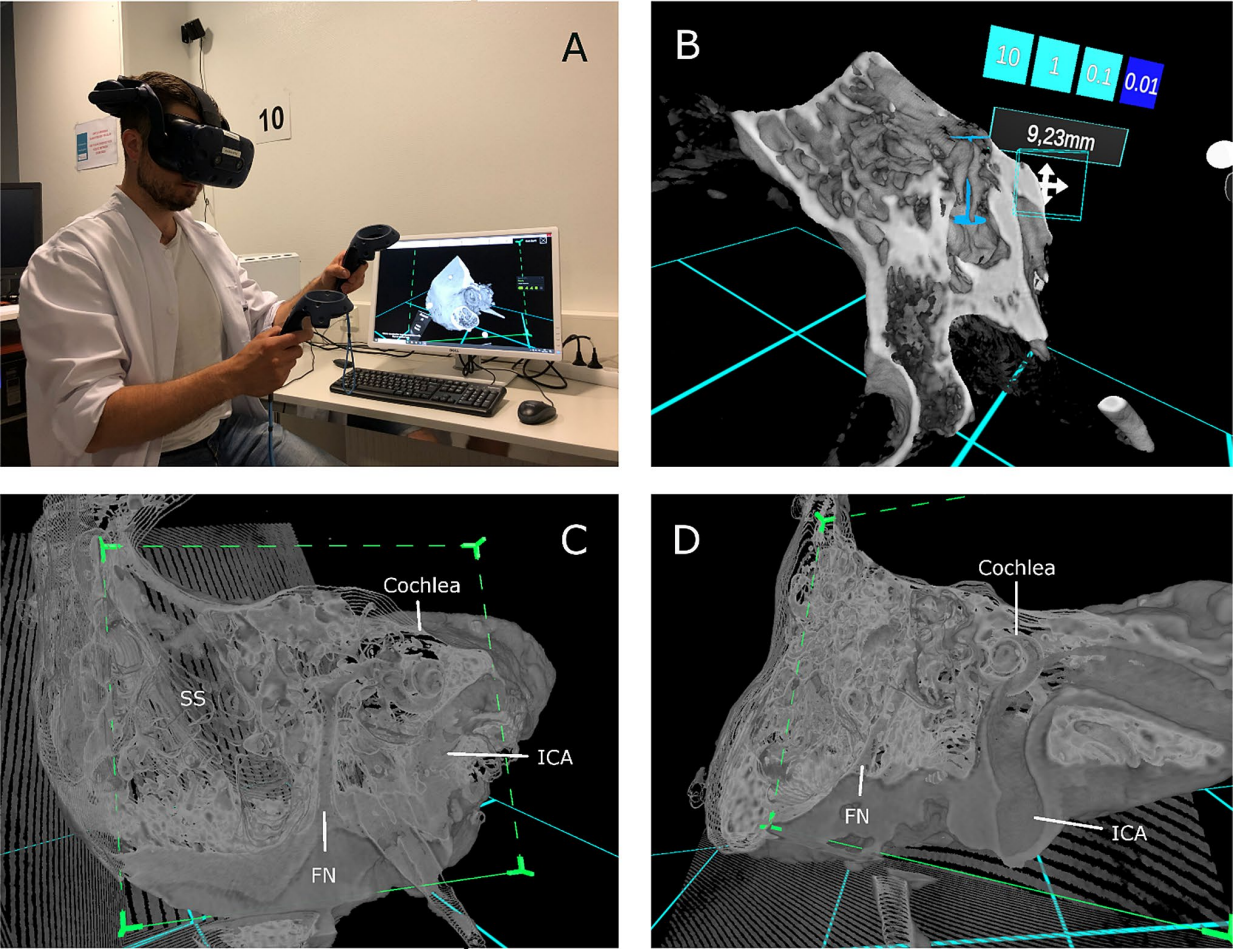
**a** VR interface during the measurement of temporal bone, **b** measurement of malleus in VR environment, **c** temporal bone in VR environment, **d** temporal bone in VR environment. *SS* Sigmoid sinus, *FN* Facial nerve, *ICA* Internal carotid artery

Five anatomically normal TBs were harvested, and five 3-mm titanium fiducial marker screws were placed on each TB to predefined locations: two at the squamous part, one at the mastoid tip and two at the petrous part (Fig. 1a). Direct physical measurement (DPM) of the distances between screw fiducials were obtained with a standard Vernier caliper (accuracy 0.02 mm) under an operating microscope by one expert otologic surgeon. DPMs were later compared with the distance measurements performed in the VR and PACS interface. In addition, 11 measurements of surgically significant anatomical structures (Table 1) such as the size of facial recess and the diameters of the oval and round windows were obtained for every TB in the VR environment and with PACS interface (Figs. 1b, c and 2b). Since DPMs for anatomical structures were not available, the measurements were compared as a percentage difference to the respective median of the anatomical measurements conducted by the five subjects.

**Table 1:** Measured anatomical distances and their clinical relevance

Anatomical measurements	Indication for measured distance:
Length of malleus (manubrium + head)	Universal measurement, easy to compare, no direct surgical relevance
Distance from body of incus to mastoid cortex	The estimate for drilling depth, e.g. in mastoidectomy
Horizontal diameter of bony ear canal (external meatus)	Estimation of drilling work during canaloplasty
Vertical diameter of bony ear canal (internal meatus)	Estimation of tympanic membrane diameter, universal anatomic measurement
Size of facial recess	Space for posterior tympanotomy
Distance from facial recess to mastoid cortex	The estimate for drilling depth regarding the facial nerve
Distance from facial nerve (mastoid part) to recess of bony ear canal	Limit of posterior drilling in canaloplasty
Diameter of oval window	Stapedotomy, stapedectomy. Identification of the oval window area and surgical access to stapes footplate
Diameter of round window	Cochlear implantation and active middle ear implantation. Identification of round window area and evaluation of surgical approach
Length of styloid process	Universal anatomical measurement, stylalgia
Distance from sigmoid sinus to back wall of bony ear canal	The space available for drilling in e.g. mastoidectomy

High resolution CT (HRCT) images were acquired using Siemens SOMATOM Definition Flash (Siemens Healthcare, Forchheim, Germany). The imaging parameters for the scans were 120 kV, 96 mAs, FOV 85 mm, pitch 0.8, CTDIvol 21.1 mGy and slice thickness of 0.4 mm. An experienced neuroradiologist and two experienced surgeons evaluated the quality of the HRCT images both in the PACS interface with conventional cross-sectional viewing (PACS) and in the VR environment (VR).

The Adesante SurgeryVision™ (Adesante Oy, Turku, Finland) medical software was used to create the VR environment and the 3D model from the HRCT data. A Headset (HTC Vive Pro, HTC, New Taipei, Taiwan) and a pair of controllers were used to interact and visualize the models in the VR environment created by the software (Figs. 1d and 2a). The cross-sectional HRCT image visualization was performed in the PACS interface (Sectra AB, Linkoping, Sweden).

### Subjects

Five experienced otologic surgeons volunteered for the study. The mean for working years in the field of ENT was 16.5 years (range 5.5–34 years). The mean for otological operations performed per year was 72 cases (range 48–120). The three less experienced subjects had performed 396–720 and the two more experienced subjects 1632–2520 otological operations as a primary surgeon. None of the subjects had any prior experience with VR.

### Tasks

Each subject received a 15-min hands-on instruction for using the VR software. The measurement tasks (see Online Resource 1.) of the fiducials and anatomical landmarks were performed in a predetermined sequence. Three preset windows were optimized to the VR for bone, soft tissue and a translucent bone visualization. In addition, the subjects were allowed to control and adjust the settings as they preferred. All five TBs were measured in randomized order. The corresponding measurements in the PACS interface were performed after a minimum of 2 weeks’ interval (mean 175 days) and in random order. Linked multiplanar 2D reconstructions which display the axial, sagittal and coronal view were used to make the measurements with PACS interface. To obtain the optimal image sections for measurement, subjects were able to rotate the view and planes in any direction (Fig. 2c, d).

### Questionnaire

After completing the tasks, the subjects evaluated the subjective validity of both the methods using a modified five-point Likert-type survey (see Online Resource 2.). Similar surveys have been previously used in temporal bone simulator studies [18–20]. The questionnaire included 20 domains in which one represented *not true/realistic/useful* and five represented *very true/realistic/useful.* A score of 3 was considered neutral. A free text section was added for specific comments on the perceived advantages and problems. The questionnaire was divided into face (FV) and content validity (CV) and global rating (GR) subgroups assessing user experience, the quality and veracity of the image illustration and hardware configuration between the methods, the level of surgical planning effectiveness and anatomy learning, and the applicability of the methods into clinical use, respectively.

### Statistical analysis

The statistical analysis was performed under the consultation of a statistician. All the statistic tests were performed with IBM SPSS statistics version 25 (IBM SPSS, SPSS Inc., Chicago, IL, USA). The level of significance was set to *p* < 0.05 for all statistical methods used in this study.

Intraclass correlation coefficient (ICC) was used as a measure of correlation between the distances measured with different methods, i.e. between VR, PACS and DPM. The inter-rater reliability describing the agreement between different subjects was defined separately for VR and PACS measurements of fiducial and anatomical distances. The anatomical distance measurements were converted and evaluated as a percentage difference compared to the median of each measurement point.

The two-tailed paired sample *t*-test and Bland–Altman analysis were performed to evaluate the equivalence of the PACS and VR measurements [21]. A linear mixed model was used to consider the possible measurement bias between different subjects. For box plots, the measurements 1.5 IQRs above the upper and below the lower quartiles were determined as outliers. Wilcoxon signed rank test was conducted for the Likert-type questionnaire statistical analysis.

## Results

All subjects completed all the given tasks. Due to a partially fractured bony ear canal in one TB eleven anatomical measures (11/275, 4%) could not be determined. The missing measurements were not method dependable (PACS data 6/11, VR data 5/11). A summary of all measurements and statistics are presented in Tables 2 and 3.

**Table 2:** Statistics in screw fiducial distance measurements obtained in conventional cross-sectional view (PACS) and in the VR environment

Subject	Method	Mean difference to DPM [mm]	SD [mm]	95% CI for mean difference [mm]	Mean difference between PACS and VR [mm]^a^	ICC PACS vs. VR	ICC PACS vs. DPM	ICC VR vs. DPM
1	PACS	− 0.291	1.344	− 0,673; 0.091	− 0.076 (*p* = 0.688)	0.995	0.995	0.998
	VR	− 0.215	0.986	− 0.495; 0.065				
2	PACS	− 0.181	3.187	− 1.087; 0.724	− 0.010 (*p* = 0.982)	0.973	0.974	0.998
	VR	− 0.171	0.895	− 0.425; 0.083				
3	PACS	− 1.129	5.295	− 2.634; 0.375	− 0.565 (*p* = 0.442)	0.933	0.929	0.998
	VR	− 0.565	0.852	− 0.807; − 0.323				
4	PACS	2.563	5.524	0.993; 4.133	3.371 (*p* < 0.001*)	0.927	0.916	0.997
	VR	− 0.808	1.066	− 1.111; − 0.505				
5	PACS	− 0.591	1.198	− 0.932; − 0.251	− 0.361 (*p* = 0.005*)	0.998	0.996	0.998
	VR	− 0.230	0.938	− 0.497; 0.036				
Total^b^	PACS	1.753	3.563	1.178; 2.327	0.472 (*p* = 0.065)	0.965 (mean)	0.962 (mean)	0.998 (mean)
	VR	0.815	0.665	0.707; 0.922				

*SD* standard deviation, *CI* confidence interval, *ICC* intraclass correlation coefficient (*p* < 0.001), *PACS* conventional cross-sectional (2D) method, *VR* virtual reality environment, *DPM* direct physical measurements by Vernier caliper

^a^Paired sample *t*-test

^b^Absolute measurement values used

^*^Statistically significant difference between the methods

**Table 3:** Statistics in anatomical distance measurements obtained in the conventional cross-sectional view (PACS) and in the VR environment

Subject	Method	Mean difference [%]	SD [%]	95% CI for mean difference [%]	Mean difference between PACS and VR [%] ^a^	ICC PACS vs. VR
1	PACS	− 0.198	11.791	− 3.386; 2.989	2.825 (*p* = 0.186)	0.978
	VR	− 3.023	16.621	− 7.517; 1.470		
2	PACS	4.130	18.650	− 0.912; 9.172	1.980 (*p* = 0.519)	0.923
	VR	2.113	16.075	− 2.232; 6.495		
3	PACS	− 3.097	22.239	− 9.109; 2.915	− 4.864 (*p* = 0.174)	0.936
	VR	1.915	20.863	− 3.690; 7.591		
4	PACS	− 2.988	59.020	− 18.943; 12.967	11.054 (*p* = 0.268)	0.900
	VR	− 14.137	39.732	− 24.878; − 3.396		
5	PACS	2.645	16.863	− 1.914; 7.203	0.300 (*p* = 0.971)	0.971
	VR	2.388	13.900	− 1.379; 6.146		
Total ^b^	PACS	12.824	27.451	9.565; 16.083	1.923 (*p* = 0.396)	0.942 (mean)
	VR	13.140	20.238	10.737; 15.542		

Measurements evaluated as a percentage difference compared to the median.

*SD* standard deviation, *CI* confidence interval, *ICC* intraclass correlation coefficient (*p* < 0.001), *PACS* conventional cross-sectional (2D) method, *VR* virtual reality environment

^a^Paired sample *t*-test

^b^Absolute measurement values used

**Fig. 3:**
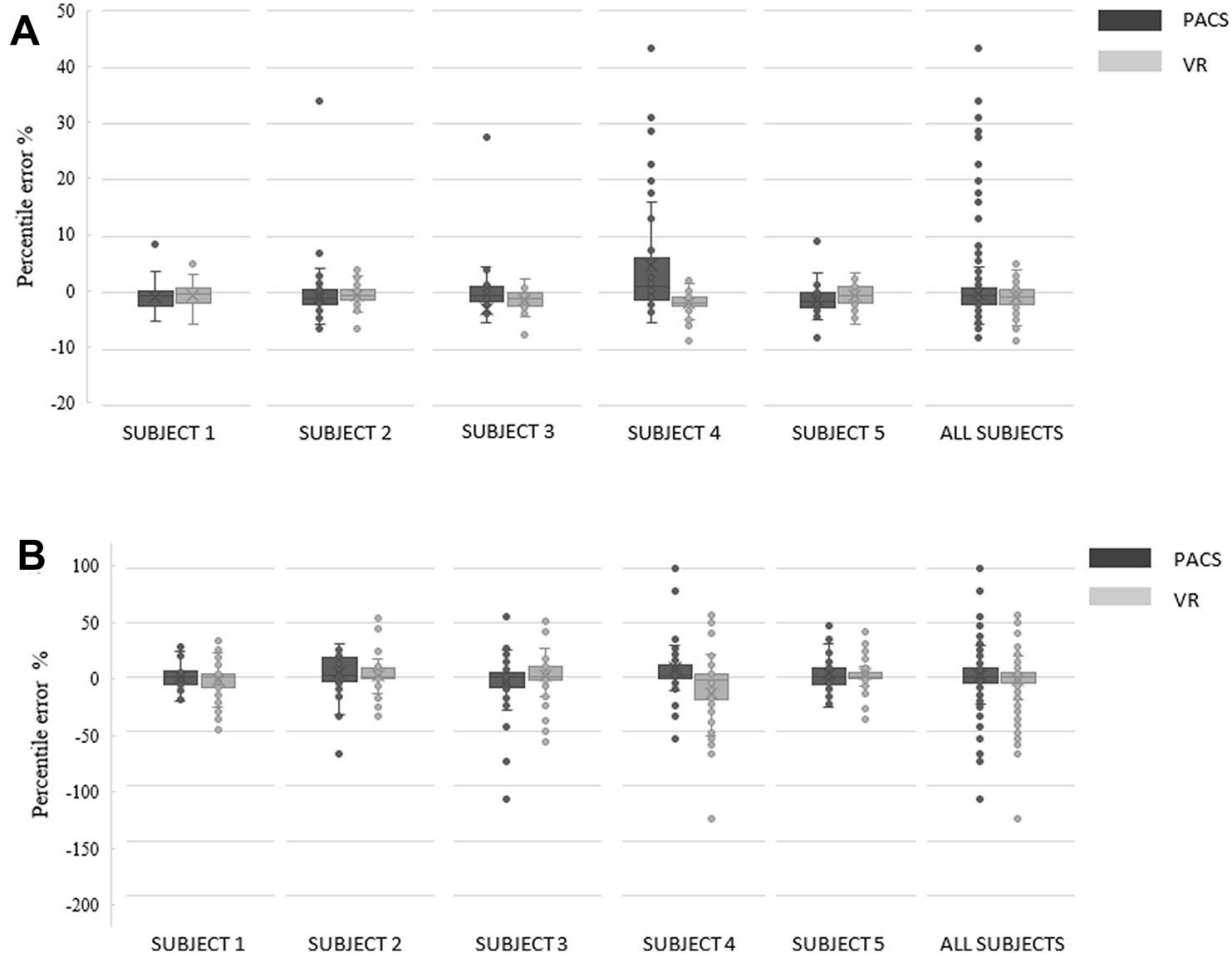
Box plots for the comparison of **a** fiducial (percentile error compared to DPMs) and **b** anatomical (percentile error compared to median of each anatomical measurement point) measurement accuracy from all subjects

The box plots illustrating the distribution of the measurements are presented in Fig. 3. The statistical data outliers were interpreted as measurement errors. Every subject made measurement errors with both methods. The total number of errors in the anatomical measurements were 58 in VR data and 27 in PACS data (Fig. 3b). The errors in the fiducial measurements were more frequent (26 vs 5) (Fig. 3a) and the mean difference to DPM was higher in PACS data than in VR data (Table 2). Of the 26 errors made in the cross-sectional viewing, 14 (54%) were most likely due to a misidentification of the fiducial measurement points (measured distance matched to a distance between other, not aimed, fiducial pair). None of the measurement errors in VR suited for fiducial misidentification. Subject 4 made several incorrect identifications of the fiducials (seven identification mistakes) in the cross-sectional viewing leading to substantial measurement errors (13 data point outliers in total), but misidentifications were also made by the more experienced subjects. There was no difference in the number of measurement errors in total between less or more experienced otologic surgeons (mean 23.7 vs. 23.0 outliers) or between methods used by less or more experienced subjects (PACS data 10.3 vs. 11.0, VR data 13.3 vs. 12.0), respectively. Thus, in this study the level of surgical experience appeared not to have an impact on the number of measurement errors in either method.

A strong correlation between the methods was found for the measurements of the fiducial (ICC ≥ 0.916; Table 2) and of the anatomical distances (ICC ≥ 0.900; Table 3). The inter-rater reliability was high in both PACS and VR methods: in the fiducial distance measurements, the mean ICC between the subjects was 0.930 and 0.999 for PACS data and VR data, respectively. Accordingly, in the anatomical distance measurements, the respective mean ICCs were 0.914 and 0.955 for PACS data and VR data.

There were no statistically significant differences in the fiducial (*p* = 0.065) and anatomical landmark measurements (*p* = 0.396) between PACS and VR data (Tables 2 and 3). However, when performing the paired sample t-test separately for each subject, we found a significant difference between the methods in two subjects in the fiducial distance measurements (Table 2). Subject 4 made significant mistakes in identification of the fiducials in the PACS interface, which explains this finding. In the contrary, the excellent measurement agreement of subject 5 resulted in a very low standard deviation and narrow confidence interval (Fig. 3, Table 2), which explains the statistical significance.

**Fig. 4:**
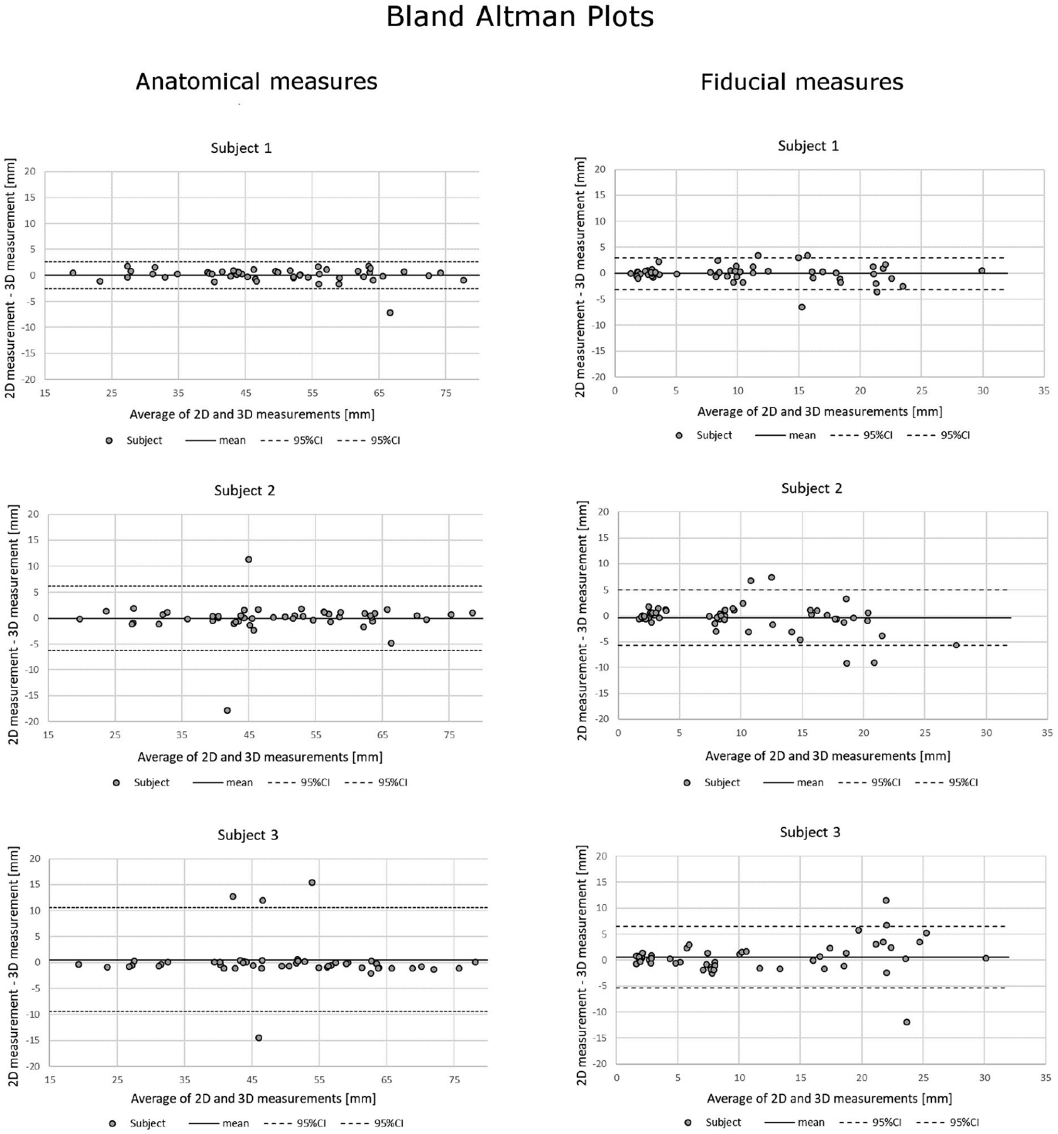

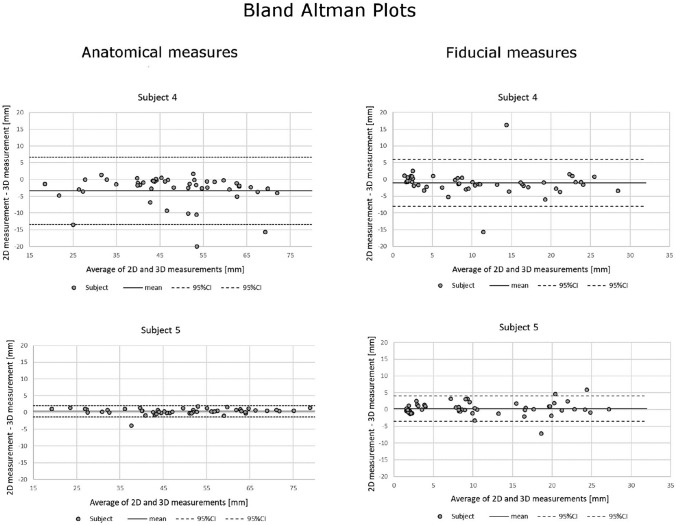
Bland–Altman plots for the comparison of anatomical measurements and fiducial measurements from all subjects

The Bland–Altman plots are presented in Fig. 4. The differences for PACS and VR data in both anatomical and fiducial measurements fell mainly within the limits of agreement for every subject. There was no systematic bias between the two methods.

All subjects considered VR helpful for the assessment of TB anatomy. Mean overall Likert scores for VR were significantly better compared to PACS scores (*p* < 0.001). In subgroup analysis, Likert scores were significantly better towards the VR for FV (*p* = 0.002) and CV (*p* < 0.001). The GR subgroup questions showed a tendency in favor of the VR method, but the difference remained statistically insignificant (*p* = 0.132) (Table 4). The analysis of the individual questions of the survey revealed statistically significant differences between the two methods presented in Table 5. The appearance of the anatomical structures, the depth perception, the understanding of the anatomical structures (e.g. the cochlea rotation within the labyrinth), the hand–eye coordination (e.g. how easy it is to grasp, move and follow the objects) and overall score for the understanding of the surgical site (e.g. in which angle to approach to round window or the risk for facial nerve damage in posterior tympanomy) in VR were significantly preferred over PACS. The mean scores for ergonomics (3.2 vs 3.6) and the subjects grade for feasibility and inclusion of the method to clinical surgical planning use (4.6 vs 4.8) were the only questions that were in favor of PACS but statistical significance (*p* = 0.317) could not be presented.

**Table 4:** Wilcoxon signed rank test for the 5-point Likert questionnaire

Questionnaire subgroup	Method	Mean score	SD	Diff. (95% CI)	*p* value
FV	PACS	3.20	0.99	0.58 (− 1.00; 0.00)	0.002*
	VR	3.78	0.83		
CV	PACS	3.23	0.62	0.9 (− 1.50; 1.00)	< 0.001*
	VR	4.33	0.62		
GR	PACS	4.10	0.85	0.25 (− 0.50 ;0.00)	0.132
	VR	4.35	0.75		
Total	PACS	3.39	0.90	0.72 (0.50; 1.00)	< 0.001*
	VR	4.11	0.78		

*FV* Face validation, *CV* Content validation, *GR* Global rating, *SD* standard deviation, *VR* virtual reality environment, *PACS* conventional cross-sectional (2D) method

^*^Statistically significant difference between the methods

**Table 5:** Wilcoxon signed rank test for individual questions of the 5-point Likert questionnaire

	VR (SD)	PACS (SD)	*p* value
FV subgroup			
Appearance of anatomical structures	4.6 (0.5)	3.4 (0.5)	0.034*
Appearance of tools	3.2 (0.4)	3.2 (0.7)	1.000
Usability of tools	3.0 (0.6)	3.0 (1.1)	1.000
Performance of tools	3.8 (0.4)	3.4 (0.5)	0.157
Haptic feedback	3.6 (0.5)	3.0 (0.6)	0.830
Ergonomics	3.2 (0.4)	3.6 (1.0)	0.317
Depth perception	4.4 (0.8)	2.0 (0.9)	0.034*
Quality of graphics	4.4 (0.8)	4.0 (0.9)	0.317
CV subgroup			
Learning of anatomy	4.6 (0.5)	3.2 (0.4)	0.059
Learning of surgical planning	4.6 (0.5)	3.4 (0.5)	0.063
Understanding of anatomical structures	4.4 (0.5)	3.4 (0.5)	0.034*
Quality of measuring anatomical structures	4.4 (0.5)	2.8 (0.7)	0.059
Understanding the relationships of anatomical structures	4.6 (0.5)	3.0 (0.6)	0.180
Accuracy of measurement tool	4.0 (0.9)	3.6 (0.8)	0.577
Hand-eye-coordination	3.8 (0.4)	3.0 (0.0)	0.046*
Overall score for surgical planning	4.2 (0.4)	3.4 (0.5)	0.102
GR subgroup			
Recommend to colleague	4.6 (0.5)	4.2 (0.7)	0.157
User-friendly	3.6 (0.8)	3.6 (1.0)	1.000
Inclusion to surgical planning	4.6 (0.5)	4.8 (0.4)	0.317
Understanding of the surgical site	4.6 (0.5)	3.8 (0.4)	0.046*

Subjects (*n* = 5)

*VR* virtual reality environment, *PACS* conventional cross-sectional (2D) method. *FV* Face validation, *CV* Content validation, *GR* Global rating, *SD* standard deviation

^*^Statistically significant difference between the methods

The analysis of the free text feedback from the questionnaire indicated that VR gave better 3D understanding of the anatomical structures and their relationship. The possibility to approach the target in any angle and direction was appreciated. All subjects valued the VR as an excellent tool for teaching anatomy and for training otologic surgery. None of the subjects reported VR to cause vertigo, nausea or headache.

## Discussion

Several studies, especially in reconstructive surgery, have demonstrated, that virtual surgical planning may improve surgical outcomes and accuracy [22, 23]. For TB anatomy specifically, few studies have evaluated virtual temporal bone dissection software for training purposes [24–26]. In a case-specific study of VR temporal bone surgery simulator 75% of users, comprising trainers and trainees, valuated VR useful for preoperative planning [27]. To the best of our knowledge, there are no clinical studies investigating the measurement accuracy of VR for TB anatomy and simulated surgical planning with expert otosurgeons. In the present study, we explored the feasibility, measurement accuracy and the subjective validity of VR planning in simulated setting with TBs and compared it to the planning in the PACS interface with standard cross-sectional viewing.

The five test subjects were experienced otologic surgeons of a tertiary center with an expert-level knowledge of temporal bone anatomy and surgical planning in the PACS interface but no prior experience with consumer or medical VR environments. The results support our hypothesis with respect to the feasibility and measurement accuracy of the used VR software of complex anatomic models such as TBs compared to cross-sectional viewing. More specifically, we found strong correlations and agreement between all distance measurements for each method (Tables 2 and 3; Fig. 4). In fact, the distance measurements in the VR environment correlated stronger with the DPM compared to those obtained in PACS interface. Similar strong correlations between VR and corresponding physical measurements have been established with previous study [13].

Since the DPMs were not available for the anatomical measurements only the correlation between both methods could be investigated. The variation in the anatomical measurements were higher in the VR than in the cross-sectional viewing (Table 3; Fig. 3b) but both methods demonstrated strong correlation.

Interestingly, in the cross-sectional viewing 14 fiducial distances were misidentified by the subjects, whereas no identification errors were made in the VR. This also contributed to the lower standard deviation for the VR measurements (Table 2), which, however, remained statistically insignificant. In addition, the measurements of anatomical landmark distances were comparable between the methods. This finding is rather compelling considering its possible clinical implications for surgical planning. It suggests that VR environment may offer more measurement accuracy of complex anatomical structures with less measurement and identification errors even for experienced surgeons. This is in accordance to previous clinical studies mainly done in reconstructive surgery, which have shown that VR planning improve the accuracy of the surgical outcomes [22, 23, 28–31].

All subjects favored VR over cross-sectional viewing in PACS interface for a more detailed apprehension of anatomy and topography. VR was also regarded beneficial for learning complex anatomy and surgical planning. VR environment allowed more detailed definition and measurements of surgically relevant spaces and distances compared to cross-sectional viewing. For example, the size of facial recess, diameter of round window were found much more assessable in VR environment. In addition, topographical relations can be better perceived in the VR environment which clinically can help to conceive, e.g. the insertion trajectory of a cochlear implant electrode.

The measurement tasks were also reported to be easier with the VR (Fig. 3). The subjects commonly reported that the accurate identification of the fiducials and the related measurements were difficult in the cross-sectional viewing. Especially, the alignment of the fiducials was considered more difficult, although the objective to use fiducial markers was to obtain fixed measurement points to mitigate the marginal for measurement errors. As mentioned earlier, all experienced surgeons misidentified some fiducials more frequently in PACS interface than in VR. This underlines our main finding that the VR environment provides more accessible information in complex anatomic situations, which is in accordance to previous studies [32–34]. There are significant differences in spatial comprehension between individuals, which affect their ability to perform an anatomical task [35, 36]. Surgeons too, including also the most experienced, are subject to the same variations in the ability to reconstruct volumetric data into a complex three-dimensional model in their mind. This is a challenging process especially for novice surgeons but may mislead also experienced surgeons like our results showed.

Some adverse effects have been previously described with the VR application use [37]. The use of head-mounted displays has been associated with nausea, oculomotor disturbances, disorientation and headache [38, 39]. In this study none of the subjects reported any adverse effects. The limited number of subjects and the diminutive need for quick turns of gaze and head in VR environment possibly caused low sensory mismatch not strong enough to generate these adverse effects.

Several strengths and limitations of this study require attention. The 5-point Likert questionnaire was limited for subjective evaluation, which is a common limitation with self-reported validation surveys. The number of test subjects was limited with different levels of otology and skull base experience, which may have confounded the results. Although not statistically significant, less experience appeared to negatively affect cross-sectional viewing. This is not very surprising since an unambiguous allocation of the fiducials or anatomic structures is easier for the visualization in VR. Since these mistakes occurred also to the most experienced surgeons, it can be doubted whether a larger number of subjects would have changed the present results. The relatively large number of measurements (525 per subject) reduced the effect of individual errors and added to the statistical power of this study and could substantiate the objective validity of the VR software for surgical planning.

This study concentrated to test the measurement accuracy of VR environment developed for surgical planning compared to conventional cross-sectional image viewing and distance measurements without temporal dissection or clinical surgery. To test the VR environment in mastoidectomy for evaluation of its feasibility in the clinical setting warrants for further studies. 3D reconstructions could also be visualized on a 2D monitor. Conventional calibrated high-quality radiological monitors may have better image quality, especially for surface and bone anatomy, and accessibility in hospitals than the current VR systems. However, VR visualization provides better stereoscopicity and immersivity than 3D reconstructions visualized in 2D monitors. The movements in virtual space are more natural and correspond better the surgical reality. Thus, it would be interesting to also compare to the 3D reconstructions on 2D monitors, conventional 2D cross-sectional viewing and VR for surgical planning in the future.

The development of more advanced VR environments and augmented reality (AR) environments with haptic devices will have a major impact on surgical planning, training and navigation in the future [40]. The AR allows users to merge both the real and virtual environments and is a continually improving technology of high interest for further studies [41]. The accurate visualization of complex anatomic structures is indispensable for adequate planning. The VR environment allows surgeons to engage the patients imaging studies like the anatomy during the surgery and simulate, e.g. the access or approach for a given patient. As the field of surgery evolves towards more patient specific and mini-invasive techniques, new techniques and tools for preoperative simulation and image viewing is needed. This study demonstrated that the accuracy of the VR environment for the assessment of TB anatomy is at least comparable to conventional cross-sectional viewing, but with the added benefit of providing more comprehensive information on topographical and spatial relation of anatomic structures. For the less experienced, the VR environment may represent an even more important tool for learning complex anatomy. Therefore, it is also important to investigate the usefulness of the VR environment for learning TB anatomy in medical students and novice otologic surgeons.

## Conclusion

The present study demonstrated the feasibility of VR for simulated surgical planning in TBs. The VR environment provided comparable results with less measurement errors than conventional cross-sectional viewing, confirming its feasibility in clinical image viewing for simulated surgical planning and adding an in-depth apprehension of complex anatomy. In addition, VR demonstrated better face and content validity compared to PACS, confirming its subjective validity. Further studies are needed to establish and confirm the objective validity and the effect of the VR surgical planning on clinical surgical outcomes in otology.

## Electronic supplementary material

Below is the link to the electronic supplementary material.

Supplementary file1 Online Resource 1. Form that lists all the measurement tasks (PDF 96 kb)

Supplementary file2 Online Resource 2. Form used for 5-point Likert validation questionnaire (PDF 153 kb)
