# Supplementary material for: Virtual reality improves the accuracy of simulated preoperative planning in temporal bones: a feasibility and validation study
Source: Eur Arch Otorhinolaryngol. 2020 Sep 22;278(8):2795–806. doi: 10.1007/s00405-020-06360-6 (PMC8266780; doi:10.1007/s00405-020-06360-6)
Supplement: Supplementary file 1 — Supplementary file1 Online Resource 1. Form that lists all the measurement tasks (PDF 96 kb) [file 405_2020_6360_MOESM1_ESM.pdf]

Electronic Supplementary Material 1.

Measurement task form

Subject number:

Date:

Temporal bone number:

	VR (mm)	PACS (mm)
SCREW FIDUCIAL MEASURES: (A=Anterior, P=Posterior, T=Tip, C= Caudal)		
Cortex A – Cortex P		
Cortex A – Cortex T		
Cortex A – Petrous A		
Cortex A – Petrous C		
Cortex P – Cortex T		
Cortex P – Petrous A		
Cortex P – Petrous C		
Cortex T – Petrous A		
Cortex T – Petrous C		
Petrous A – Petrous C		
ANATOMICAL MEASURES:		
Length of malleus (manubrium+head)		
Distance from body of incus to mastoid cortex		
Horizontal diameter of bony ear canal (external meatus)		
Vertical diameter of bony ear canal (internal meatus)		
Size of facial recess		
Distance from facial recess to mastoid cortex		
Distance from facial nerve (mastoid part) to recess of bony ear canal		
Diameter of oval window		
Diameter of round window		
Length of styloid process		
Distance from sigmoid sinus to back wall of bony ear canal		
