# Supplementary material for: Virtual reality improves the accuracy of simulated preoperative planning in temporal bones: a feasibility and validation study
Source: Eur Arch Otorhinolaryngol. 2020 Sep 22;278(8):2795–806. doi: 10.1007/s00405-020-06360-6 (PMC8266780; doi:10.1007/s00405-020-06360-6)
Supplement: Supplementary file 2 — Supplementary file2 Online Resource 2. Form used for 5-point Likert validation questionnaire (PDF 153 kb) [file 405_2020_6360_MOESM2_ESM.pdf]

Electronic Supplementary Material 2.

Validation questionnaire for conventional 2D method (PACS) and VR environment (VR)

Grading scale 1-5

1=not true/realistic/useful
2=somewhat not true / not realistic / not useful
3=neutral
4=somewhat true / realistic / useful
5=very true / realistic / useful

**Face validation subgroup (FV):**	PACS	VR
Appearance of anatomical structures		
Appearance of tools		
Usability of tools		
Performance of tools		
Haptic feedback		
Ergonomics		
Depth perception		
Quality of graphics		
**Content validation subgroup (CV):**		
Learning of anatomy		
Learning of surgical planning		
Understanding of anatomical structures		
Quality of measuring anatomical structures		
Understanding the topography and relationships of anatomical structures		
Accuracy of measurement tool		
Hand-eye-coordination		
Overall score for surgical planning		
**Global rating subgroup (GR):**		
Recommend to colleague		
User-friendly		
Inclusion to surgical planning		
Understanding of the surgical site		

Free response section:

1. Did you experience nausea/vertigo/headache or any other similar problems during the use of VR application?
2. What kind of problems did you experienced in VR surgical planning? In PACS method?
3. What would be an adequate time for orientation of the VR equipment?
4. What kind of benefits you consider the VR environment has compared to PACS method in surgical planning and in the understanding of topographical anatomy of objects?
5. In what other applications VR environment could be applied (e.g. education/surgical training)
6. Free feedback
